# Interplay Between Vertical and Horizontal Schemes of Computation: From Bayesian Inference to Quantum Logic via Gluing Boolean Algebras

**DOI:** 10.3390/e28050498

**Published:** 2026-04-28

**Authors:** Yukio-Pegio Gunji, Kyoko Nakamura, Kazuto Sasai, Iori Tani, Mayo Kuroki, Alessandro Chiolerio, Andrew Adamatzky, Andrei Khrennikov

**Affiliations:** 1 Department of Intermedia Art and Science, School of Fundamental Science and Engineering, Waseda University, 3-4-1 Ohkubo, Shinjuku-ku, Tokyo 169-8555, Japan; 2 Department of Fine and Applied Arts, Kyoto University of the Arts, 2-116 Uryuyama Kitashirakawa, Sakyo-ku, Kyoto 606-8271, Japan; kyoko608@gmail.com; 3 Department of Computer and Information Sciences, College of Engineering, Ibaraki University, 4-12-1 Nakanarusawa-cho, Hitachi 316-8511, Japan; kazuto.sasai.z@vc.ibaraki.ac.jp; 4 Information Science and Technology Center, Kobe University, 1-1 Rokkodai-cho, Nada-ku, Kobe 657-8501, Japan; iori_tani@turquoise.kobe-u.ac.jp; 5 Waseda Research Institute for Science and Engineering, 3-4-1 Ohkubo, Shinjuku-ku, Tokyo 169-8555, Japan; m.kuroki.358@gmail.com; 6 Bioinspired Soft Robotics, Istituto Italiano di Tecnologia, Via Morego, 30, 16163 Genova, Italy; alessandro.chiolerio@gmail.com; 7 Unconventional Computing Laboratory, University of the West of England, Coldharbour Lane, Bristol BS16 1QY, UK; andrew.adamatzky@uwe.ac.uk; 8 Department of Mathematics, Linnaeus University, Universitetsplatsen-1, 352 52 Växjö, Sweden; andrei.khrennikov@lnu.se

**Keywords:** material, computing, quantum logic, Bayesian inference, cause–effect relation

## Abstract

Artificial intelligence is typically formulated as an information-processing system composed of artificial neurons, where computation is understood as recursive operations connecting inputs and outputs. However, real neural systems are materially embodied and continuously reconfigured by metabolic and physical processes, suggesting that computation cannot be reduced to fixed causal structures. In this paper, we propose a theoretical framework that captures the interplay between informational and material processes as the interaction between two computational schemes: a vertical scheme, representing fixed cause–effect relations, and a horizontal scheme, representing transformations between such relations. We show that the vertical scheme corresponds to Bayesian inference, which updates probability distributions over a fixed hypothesis space, and is consistent with the free-energy minimization principle. In contrast, the horizontal scheme is formalized as inverse Bayesian inference, which modifies the hypothesis space itself by updating likelihood structures based on experienced data. We further demonstrate that the interplay between these schemes can be expressed algebraically as a process of continuously gluing Boolean algebras. This construction yields a non-distributive orthomodular lattice, i.e., quantum logic, without invoking Hilbert space formalism. In this view, quantum logic emerges not as a static logical system but as a structural consequence of dynamically reconfiguring causal contexts. This framework provides a unified perspective in which inference is understood not only as optimization within a fixed model but also as a process that generates and transforms the model itself. It offers a formal basis for describing open-ended computation and suggests a connection to approaches such as unconventional computing and Natural Born Intelligence, where computational structures evolve through interaction with material processes. Unlike existing approaches, this framework derives quantum-logic-like structure from the continual reconfiguration of causal contexts rather than from Hilbert-space assumptions or optimization within a fixed hypothesis space.

## 1. Introduction

Artificial neurons that constitute AI are defined as operations that connect the input and output of information, and the entire system is defined as a parallel distributed processing system composed of artificial neurons [1,2,3]. Scientific theories have often succeeded by adopting the method of first approximation, which simplifies and abstracts reality. The rise of AI is undoubtedly another example of this [1,2].

However, this assumption neglects the fact that real neurons have the body [4,5,6,7], are composed of water, proteins, and various organic substances, and that they metabolize and live [8,9]. Neural activity is deeply constrained and continuously reconfigured by metabolic, vascular, glial, and immune processes, indicating that the brain cannot be adequately modeled as an information-processing network [10,11,12,13,14,15,16,17,18].

For instance, during sleep, cerebrospinal fluid influx expands the extracellular space by up to 60 percentage, fundamentally altering the physical boundary conditions of neuronal interactions [19,20,21]. This suggests that neural computation cannot be understood solely as information processing on a fixed network, but must be viewed as a dynamically reconfiguring system embedded in metabolic and fluid processes [22,23].

Especially, this simplification in artificial neurons may overlook an important issue concerning creativity. Boden classified creativity into three stages: Combinational creativity, Exploratory creativity, and Transformational creativity [24]. While she argued that the last one is essential, she also pointed out that the linguistic space in which creativity is realized is itself connected to the real world, including context and the material world. Because of this connection, even a single word can generate new meaning, and creativity emerges precisely there. This suggests that creativity appears at the interface between the world of language (the world of information) and the world of matter. Ignoring the relation between neurons as information systems and neurons as material systems therefore amounts to ignoring creativity, which is one of the most essential aspects of intelligence [4,5,8,9].

These observations suggest that neural systems cannot be adequately described by inference within a fixed computational structure. Instead, the underlying causal relations themselves may be continuously reshaped by ongoing material processes. This raises a fundamental question: how can computation be formalised when both the inference mechanism and the space of possible models are subject to transformation? In this paper, we address this question by introducing a conceptual and mathematical distinction between two complementary modes of computation. The first, which we call the vertical scheme, captures inference within a fixed set of cause–effect relations. The second, the horizontal scheme, captures transformations between such relations, corresponding to changes in the underlying model or hypothesis space. By formalising the interaction between these two schemes, we aim to provide a framework in which computation is understood not only as optimisation within a given structure, but as a process that continually generates and reorganises that structure itself.

Algebraically, a Boolean algebra corresponds to a single cause–effect relation. The process in which different Boolean algebras are successively glued together while maintaining quantum logic can be regarded as an abstraction implementing the interplay between information and matter [25,26]. When interpreted as a dynamical system, a Boolean algebra represents the logic of a dynamical system that establishes a one-to-one correspondence between experience and prediction. In this sense, it corresponds to Bayesian inference [27,28,29]. Bayesian inference is a method of reasoning in which multiple hypotheses are fixed with respect to likelihood, and the probability distribution of hypotheses is updated depending on experience. Through this process, a hypothesis consistent with reality is selected and used for decision making [27,28,29,30,31].

Therefore, the process in which different Boolean algebras are glued together implies that hypotheses themselves increase or are modified. This process includes what is called inverse Bayesian inference [32,33,34]. Thus, the process of gluing Boolean algebras while maintaining quantum logic suggests a system that simultaneously realizes Bayesian inference and inverse Bayesian inference. The present contribution differs from standard free-energy approaches in that it does not restrict inference to optimisation within a fixed hypothesis space. It differs from most quantum-cognition approaches in deriving non-classical logical structure from the continual transformation and gluing of classical contexts, rather than postulating quantum-like probabilistic structure at the outset.

The approach of obtaining quantum logic by gluing Boolean algebras without introducing Hilbert spaces or superposable probabilistic processes can be regarded as a lattice-theoretic development of the approach proposed by Abramsky and Coecke [35]. Here we further develop this idea in the context of Bayesian inference. Currently, neuroscience and AI increasingly interpret brain computation in terms of Friston’s free-energy minimization principle, which generalizes Bayesian inference as prediction error minimization [27,28,30,31,36,37,38,39]. The interplay between opposing or complementary aspects has also been studied in the context of bipolar fuzzy sets, where positive and negative components are treated simultaneously within a unified framework [40].

The present study proposes introducing a horizontal extension of computational processes in addition to the vertical direction of causal inference, and shows that this leads to quantum logic.

## 2. Vertical Scheme and Horizontal Scheme of Computation

How can the interplay between information systems and material systems be abstracted and theorized? Let us consider a neural network. A neuron represented as an information system assigns weights to many discrete values by synaptic weights, sums them, and discretizes the result with a various activation functions from threshold function [41,42,43,44,45,46,47] to produce an output. Such a system can be described by recursive functions [48,49,50] equivalent to Turing machines [51] or automata.

When many such neurons are arranged in parallel, they repeatedly perform recursive computations until reaching the output layer. These processes do not go beyond the scope of composition of recursive functions, induction, and projection. The output values are evaluated by the environment or by the system itself, and the synaptic weights of each neuron are modified accordingly. This is learning. Even the way in which weights are modified based on outputs remains within the domain of recursive functions.

Therefore, an information system can be regarded as a system described by recursive functions. Learning produces a computational processing system, and decisions are made using the learned system. In terms of causality, learning is a reverse causal process that produces a cause (the processing system) from an effect, and thus corresponds to induction. In contrast, the process of obtaining a decision as an effect from a completed computational system as a cause corresponds to deduction. Although these two processes have opposite directions, they are both expressed as recursive functions in computation. They constitute computation in a vertical scheme that connects cause and effect.

However, real neural networks cannot be fully described as information processing systems written entirely by recursive functions. When electrical signals sent from axons are received at synapses of neurons, biochemical substances inside the cell dissociate, such as exocytosis, NMDA receptors and Ca influx [52,53,54,55]. These substances called second messengers propagate inside the cell while undergoing self-aggregation and pattern formation [56,57,58]. As a result, when they reach the synapses responsible for output, new electrical signals are emitted from those synapses [59].

Artificial neurons are obtained by simplifying biochemical reactions and diffusion processes involved in such material processes and approximating them with various activation functions or majority operations. Therefore, the material system contributes to artificial neurons in the form of parameter changes or changes in boundary conditions [60]. The contribution of material systems is not simple. Recent studies have shown that during the night, cerebrospinal fluid rises and enters the brain during sleep, literally “washing away memories” [19,20,21,22,23]. This means that the concentrations and distributions of proteins and carbohydrates inside neurons change dramatically. As a result, synaptic weights themselves and the motion of intracellular solutions change, which may alter the operation that sums inputs.

Material processes thus make significant contributions to the information system in the form of transformations of parameter spaces and state spaces. When the information system is understood as the vertical scheme of information, the contribution of the material system can be considered as a horizontal transformation of that information system, namely a transformation of causal relations that had been established solely by the information system [61].

To make this distinction precise, we introduce the following operational definitions. A vertical scheme of computation is defined as a computational process in which inference is performed within a fixed causal structure. In this setting, the set of variables, their relations, and the hypothesis space remain invariant, and computation proceeds through updating states, parameters, or probability distributions. Typical examples include recursive computation and Bayesian inference, where outputs are derived from inputs under a stable mapping. A horizontal scheme of computation, in contrast, is defined as a process that transforms the causal structure itself. Here, the space of variables, relations, or hypotheses is not fixed but is modified through interaction with data, context, or material processes. This includes operations that generate new hypotheses, alter likelihood structures, or reconfigure the mapping between inputs and outputs. In this sense, vertical schemes operate within a given model, while horizontal schemes operate on the model, enabling transitions between distinct causal organisations. The interplay between these two schemes therefore captures computation as a process that not only evaluates and updates states, but also reorganises the structure in which such evaluation takes place. This distinction will be used throughout the paper to connect Bayesian inference with vertical computation and inverse Bayesian inference with horizontal transformation.

With regard to language, the relation between information systems and material systems can be understood as the relation between a linguistic space defined by the pair of syntax and semantics, and an action space that includes the surrounding material world. Language that is believed to be determined by meaning is nothing more than a computational process that takes values, and the essence of generative grammar, which is considered a universal language, lies in recursion [62]. In this sense, the linguistic space defined by generative grammar is nothing more than recursive functions.

The surrounding action space dramatically transforms the linguistic space that is believed to be used according to meaning. In the linguistic space, the vertical scheme connecting cause (sentence) and effect (understanding) is determined by meaning. In contrast, in the action space there also exists a vertical scheme connecting cause (sentence) and effect (understanding), but what determines it is not meaning but performativity [63]. These two are not separated but can be connected through the attenuation of meaning.

For example, the liar sentence “I am a liar” is understood as a contradiction in the sense that the meaning of the effect (understanding) cannot be determined from the cause (sentence). However, the same sentence, “I am a liar”, can also be used as a password to enter a building among friends. This shows that the sentence is understood performatively through the attenuation of meaning, without being constrained by meaning [25].

**Figure 1:**
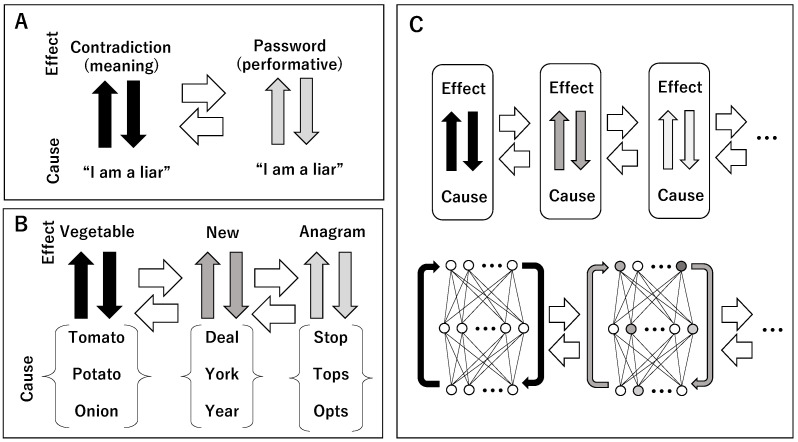
The relationship between a vertical scheme representing a cause–effect relation and a horizontal scheme representing its transformation. (**A**). An example where the expression “I am a liar” (cause) is understood (effect) from a semantic contradiction to a password based on performative-ness. (**B**). An example where the relationship between a set of words (cause) and the commonality of words (effect) changes to attributes, prefixes, and anagrams. (**C**). Generalization of the cause–effect relation and its transformation (**upper panel**) and the cause–effect relation and its transformation in neural networks.

Thus, through attenuation of meaning, one cause–effect relation can be transformed into another cause–effect relation (Figure 1A). Although the linguistic space and the action space have been described as separate, the relation between them can also be regarded as continuous. Language is not bound by fixed meanings but continuously changes its meanings through contact with the real world as an action space. The transformation of meaning suggested by Boden [24] lies precisely here. Therefore, as shown in Figure 1B, the way in which a given set of words forms a set can vary widely. For example, on the left is a set connected by semantic relations, in the middle a set of words formed by adding “New” at the beginning, and on the right a set connected by anagrams. In this way, the causal relation between a set as a cause and the understanding that it constitutes a set as an effect can change in many ways. Such transformations are obtained through the attenuation of existing cause–effect relations, and cause–effect relations become glued together. As a result, the cause (sentence or word) and the effect (understanding) in language realize a movement that freely traverses different cause–effect relations.

The process of attenuation from linguistic space to action space (which is not a discrete separation of spaces but a continuous transformation) can be reinterpreted as a process of transformation between computational processes and material processes. The schematic diagram representing this interpretation is shown in the upper part of Figure 1C. If the cause–effect relation in language (the relation between sentence and understanding) is regarded as the process of a vertical scheme, then the transformation of causal relations through connections with context, action, and the material world corresponds to a horizontal scheme. The upward arrows in the vertical direction represent the determination of understanding, and the downward arrows represent the determination of expression (sentences or words). The change in their colors horizontally from black to dark gray and light gray indicates that the methods of determining understanding and expression are being transformed.

The philosophy that considered such horizontal schemes in language was Wittgenstein’s language games [64]. In this paper, we attempt to extend language games to computation realized by matter. Applying the interplay between vertical schemes and horizontal schemes to actual neural networks yields the lower diagram of Figure 1C. Neural networks proceed from lower input layers through intermediate layers to higher output layers. In this sense, the upward arrows in the vertical direction represent decision making, and the downward arrows represent learning processes that change synaptic weights. The arrows progressing in the horizontal direction represent the transformation of neurons at the material level. As a result, the circles representing neurons change their colors to lighter or darker gray, indicating changes in the functions of the neurons themselves. For example, in artificial neurons, discrete outputs are generated using threshold functions or various activation functions (e.g., Swish [41], GELU [42], SELU [43], GLU [45,46], ACON [47]). Changing parameters such as thresholds or slopes that determine these functions corresponds to transformations of the neurons themselves.

Such transformations eventually change the meaning and strength of both decision making and learning processes. Therefore, the vertical scheme is also transformed through the process of the horizontal scheme. To represent this, the colors of the vertical arrows are also changed. Conversely, from the interplay between horizontal schemes and vertical schemes in neural networks, the upper diagram of Figure 1C can be reinterpreted as a computational process realized by matter. In this interpretation, cause–effect relations between input and output constitute the vertical scheme, while their material transformations are implemented as the horizontal scheme.

This corresponds to unconventional computing [65,66,67,68]. Thus, computation in the sense of recursive functions and the material processes that implement it can be generalized as the interplay between a vertical scheme representing fixed cause–effect relations and a horizontal scheme that transforms those relations. The question then becomes how such a structure can be expressed logically and algebraically. This is the issue to be examined next.

## 3. Quantum Logic as a Process of Continuously Gluing Boolean Algebras

As described in the previous section, the interplay between computation represented by recursive functions and material processes was conceived as the interplay between cause–effect relations represented by the vertical scheme and their transformation represented by the horizontal scheme. In this section we express this interplay as a logical structure. That is, the vertical scheme is expressed as a specific logic dependent on a particular context, and its transformation is expressed as a horizontal scheme. Such specific logics can be added indefinitely. As a result, the overall logic always possesses multiple contexts and allows transitions between them while maintaining the independence of each context. This constitutes the interplay between vertical schemes and horizontal schemes expressed as a logical structure. Moreover, it will be shown that the entire logic maintains quantum logic [26,69,70,71] even when new contexts are added.

Here we consider logic in terms of the algebraic structure called a lattice [72]. A set is given simply by distinguishing its elements. When a binary relation satisfying reflexivity, antisymmetry, and transitivity is introduced among these elements, the resulting set becomes a partially ordered set. For two elements x,y in a partially ordered set, elements greater than or equal to both are called upper bounds, and elements less than or equal to both are called lower bounds. The least upper bound and the greatest lower bound are called the join and the meet, respectively, and are denoted by x∨y and x∧y.

If a partially ordered set *L* satisfies

(1) x∨y∈L,x∧y∈L

for all x,y∈L, then *L* is called a lattice. In particular, if for all x,y,z∈L the distributive law

(2) x∧(y∨z)=(x∧y)∨(x∧z)

holds, the lattice is called a distributive lattice. Let 0 and 1 denote the least and greatest elements of the lattice *L*. If for every x∈L there exists $x^{⊥}\in L$ satisfying

(3) $$x\wedge x^{⊥}=0\;\mathrm{and}\;x\vee x^{⊥}=1,$$

(4) $$x\leq y\Rightarrow y^{⊥}\leq x^{⊥},$$

(5) $${\left(x^{⊥}\right)}^{⊥}=x,$$

then *L* is called an orthocomplemented lattice, and $x^{⊥}$ is called the orthocomplement of *x*.

A lattice that is both distributive and orthocomplemented is defined as a Boolean algebra. Furthermore, an orthocomplemented lattice *L* is called an orthomodular lattice if and only if for x,y∈L

(6) $$x\leq y\Rightarrow y=x\vee (y\wedge x^{⊥}).$$

An orthomodular lattice in which the distributive law does not hold corresponds to quantum logic. Boolean algebra is also orthomodular lattice, where it is distributive. Any finite Boolean algebra is isomorphic to the power set of a certain set. If a set *S* has *n* elements, the power set P(S) consists of all subsets of *S*, and therefore the number of its elements is

(7) $$\left|P\left(S\right)\right|=2^{n}.$$

Thus, a finite Boolean algebra is a lattice with $2^{n}$ elements for some natural number *n*. Two Boolean algebras can be glued together by identifying their least and greatest elements. The operation of creating quantum logic by gluing Boolean algebras can be defined in category theory using pushout, and the authors have shown that it is left adjoint of a forgetful functor [73].

Let two Boolean algebras be denoted by $B_{k}$(k=1,2), with least and greatest elements $0_{k}$ and $1_{k}$, respectively. We define a new lattice *L* obtained by gluing them together as follows.

(8) $$x\in L\Leftrightarrow \left\{\begin{array}{c} x\in B_{k}\;\mathrm{and}\;x\neq 1_{k},0_{k}\;\left(k=1,2\right),\\ x=0,\\ x=1. \end{array}\right.$$

where 0 and 1 denote the least and greatest elements of *L*. The order relation in *L* is also defined by

(9) $$x\leq y\;\left(\mathrm{in}\;L\right)\Leftrightarrow \left\{\begin{array}{c} \;x\leq y\;(\mathrm{in}\;B_{k}\;k=1,2),\\ x=0,\\ y=1. \end{array}\right.$$

The original Boolean algebras become sublattices of *L*, which we denote by $B_{k}^{L}$(k=1,2) and call sub-Boolean algebras.

It is clear that *L* is an orthomodular lattice. Indeed, for every x∈L with x≠0,1, the orthocomplement $x^{⊥}$ exists in the sub-Boolean algebra to which *x* belongs, and therefore *L* is an orthocomplemented lattice.

Moreover, for x,y∈L with x≤y, if x=0 then

(10) $$y=0\vee (y\wedge 0^{⊥})=y,$$

(11) $$1=x\vee (1\wedge x^{⊥})=1.$$

In the remaining cases *x* and *y* belong to the same sub-Boolean algebra $B_{k}^{L}$, and therefore

(12) $$x\leq y\Rightarrow y=x\vee (y\wedge x^{⊥})$$

holds. Hence *L* is an orthomodular lattice. Now consider x,y,z∈L such that $x,y\in B_{i}^{L}$, $z\in B_{j}^{L}$(i≠j), and neither x≤y nor y≤x holds, so that *x* and *y* form an antichain. Then

(13) x∧(y∨z)=x∧1=x,

while

(14) (x∧y)∨(x∧z)=(x∧y)∨0=x∧y≠x.

Thus the distributive law does not hold. Therefore, *L* is a non-distributive orthomodular lattice, namely quantum logic. Furthermore, additional Boolean algebras can be glued to this non-distributive orthomodular lattice by identifying their least and greatest elements. Repeating this operation produces a lattice composed of sub-Boolean algebras $B_{k}^{L}$(k=1,2,…,n).

In other words, as new Boolean algebras are successively glued together, the resulting structure continuously maintains quantum logic.

**Figure 2:**
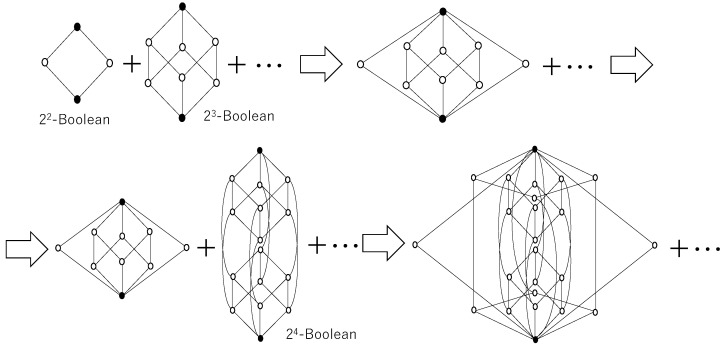
A Hasse diagram showing how a non-distributive orthomodular lattice (quantum logic) is maintained by pasting a Boolean algebra onto an orthomodular lattice, with the greatest and least elements being common elements. A Hasse diagram represents the elements of an ordered set as circles, with higher-level elements in the order relation written above and lower-level elements below, connected by lines. Since the order satisfies transitivity, lines are not drawn between two elements in the order relation if another element exists between them. However, the initial diagram is a Distributive orthomodular lattice, which is a Boolean algebra. This illustrates how multiple local causal contexts can be combined into a single, globally non-classical logical structure.

Figure 2 shows the process of sequentially gluing Boolean algebras, starting with the gluing of $2^{2}$-Boolean and $2^{3}$-Boolean algebras. Gluing the top leftmost and second-from-left lattices according to the aforementioned gluing process yields the lattice depicted in the third Hasse diagram from the left in the top row. This is an orthomodular lattice where the distributive law does not hold, and is no longer a Boolean algebra but quantum logic. When a $2^{4}$-Boolean algebra (the second Hasse diagram from the left in the bottom row of Figure 2) is further superimposed onto the quantum logic obtained in this way, the third Hasse diagram from the left in the bottom row of Figure 2 is obtained. Since different Boolean algebras are superimposed in such a way that they share only their least and largest elements, ortho-complements exist in each sub-Boolean algebra, thus preserving orthomodularity, and only the distributive law becomes invalid. In other words, quantum logic is maintained indefinitely.

Conceptually, this construction has a direct interpretation in terms of computation. Each Boolean algebra can be understood as representing a fixed causal context, corresponding to a vertical scheme of inference in which relationships between variables are stable. The gluing operation then represents the introduction of new contexts that are only partially compatible with previous ones, sharing boundary conditions but differing internally. As additional Boolean algebras are incorporated, the system no longer operates within a single global causal structure but instead traverses multiple, locally consistent structures. The resulting non-distributive orthomodular lattice therefore captures a situation in which inference is distributed across evolving contexts, rather than confined to a fixed logical space. In this sense, the emergence of quantum logic should be interpreted not as the imposition of a non-classical formalism, but as a structural consequence of continuously extending and reorganizing classical causal models through their interaction.

The coexistence of complementary or opposing elements has been formalized in bipolar fuzzy set theory [40]. This idea suggests that logical structures need not be globally consistent, but can accommodate multiple coexisting relations, which is closely related to the emergence of quantum logic as non-distributive lattices. Our approach is consistent with this perspective.

From a contextual viewpoint, Khrennikov showed that non-classical probability structures can be derived from context-dependent transformations of classical probabilities [74,75]. This suggests that quantum-like logic may arise from the composition and transformation of multiple classical contexts, which aligns with our construction of quantum logic through the gluing of Boolean algebras.

The elements shared through the gluing of Boolean algebras acquire the ambiguous characteristics of a subsystem, and in that sense, can be considered entanglement-like, unable to be allocated to a subsystem. Extending this idea, we can predict that even classical systems may exhibit behavior similar to entanglement in quantum systems. The quantum entanglement-like behavior experimentally demonstrated by [76] in magnetic fluids could be explained by such a theory.

## 4. Interplay Between Bayesian Inference and Inverse Bayesian Inference

### 4.1. General Framework

Let us reconsider the picture of quantum logic as a logic obtained by continuously gluing Boolean algebras, this time by returning to neural networks. An algebraic structure will be expressed as a dynamical system that realizes it. First, let us interpret Boolean algebra in the context of neural networks and AI. Boolean algebra is a logic that allows all phenomena to be interpreted as combinations of atoms. An atom is a unit of recognition. Such units are determined by the observer and by experience. A particular unit of recognition can be defined as the probability distribution of recognized information or data *d*. This corresponds to the probability distribution of data that are assumed to occur under a particular hypothesis *h*. When data are represented by natural numbers d=0,1,…,n, we have

(15) $$P\left(h\right)=\sum_{d=0}^{n}P\left(h,d\right),$$

and using conditional probabilities we can write

(16) $$P\left(h\right)=\sum_{d=0}^{n}P\left(h|d\right)P\left(d\right).$$

The distribution of *d* under a hypothesis *h* is called the likelihood, denoted by P(d|h). The probability distribution is normalized so that

(17) $$\sum_{d=0}^{n}P\left(d|h\right)=1.$$

If multiple hypotheses exist, h=0,…,m, then

(18) $$\sum_{h=0}^{m}P\left(h\right)=1.$$

This means that the way in which the world is understood depends on the recognition units represented by P(h). Estimating recognition units depending on experience corresponds to Bayesian inference [77,78,79,80].

If the probability of hypothesis *h* depending on experience *d* is denoted by P(h|d), then Bayesian estimation regards this as a general hypothesis independent of experience and expresses it as

(19) P(h)=P(h|d).

Once recognition units are determined depending on experience, phenomena are understood through combinations of those units. This corresponds precisely to the structure of Boolean algebra. The addition of probabilities corresponds to the join operation of Boolean algebra. Since Boolean algebras are isomorphic to power sets, the join operation corresponds to set union. Thus the sum of probabilities represents set union, and the maximal element of the Boolean algebra represents the totality of all hypotheses. Cox’s theorem [81] shows that if we assume that a proposition can be written as a Boolean algebra, we can derive a Kolmogorv-type axiomatic system of probability, thus guaranteeing the use of formulas such as P(A∧B)=P(A|B)P(B).

This Bayesian estimation constitutes the central concept of Friston’s free-energy minimization principle [27,38,39], which forms the basis of AI and neuroscience.

For simplicity, let the internal state vector of the brain be denoted by *h*, and let the stimulus given from outside the brain be denoted by *d*. The free energy is then written as

(20) $$F\left(h;d\right)=D_{KL}\left(P\left(h\right)\|P\left(h|d\right)\right)-\ln P\left(d\right).$$

Here lnP(d) represents the surprise of the data, that is, the surprise caused by experience. The quantity $D_{KL}\left(p\|q\right)$ is the Kullback–Leibler divergence, defined as

(21) $$D_{KL}\left(p\left(x_{i}\right)\|q\left(x_{i}\right)\right)=\sum_{i}p\left(x_{i}\right)\log\frac{p(x_{i})}{q(x_{i})}.$$

Clearly,

(22) $$D_{KL}\left(p\|q\right)=0\Leftrightarrow p=q.$$

Therefore, minimizing free energy,

(23) $$\min_{h,d}F\left(h;d\right),$$

means satisfying

(24) P(h)=P(h|d)

under the condition of minimizing the surprise caused by experience. This is equivalent to performing Bayesian estimation under the condition of minimizing prediction error.

From the above, the free-energy minimization principle [27,38] that underlies AI and neuroscience can be regarded as Bayesian estimation. The resulting mode of understanding phenomena therefore reduces to Boolean algebra. This corresponds to the vertical scheme of computation representing causal relations.

How, then, can the horizontal scheme of computation discussed in this paper be expressed? It can be implemented as an operation that continually adds new Boolean algebras to the distribution of hypotheses that is determined through Bayesian estimation. In fact, we have already proposed an estimation operation called inverse Bayesian inference, which implements an operation that potentially adds Boolean algebras. In Bayesian inference the hypotheses themselves possessed by the system do not change. In contrast, inverse Bayesian inference continually calculates the empirical probability of experienced data *d* and modifies the likelihood of hypotheses accordingly.

For any d=0,1,…,n, this modifies the likelihood of a particular hypothesis $h_{i}$ as

(25) $$P\left(d|h_{i}\right)=P\left(d\right),$$

where

(26) $$\sum_{d=0}^{n}P\left(d\right)=1,\;\sum_{d=0}^{n}P\left(d|h_{i}\right)=1.$$

Furthermore, for any h=0,1,…,m,

(27) $$P\left(h_{i}\right)\leq P\left(h\right).$$

Thus the likelihood of the least-used hypothesis is rewritten depending on the frequency of experienced data. Including the rewritten hypothesis, the conditional probability depending on experienced data *d* at each time is calculated as

(28) $$P\left(h|d\right)=\frac{P(d|h)P(h)}{\sum_{h}P\left(d|h\right)P\left(h\right)}.$$

Through Bayesian inference, the generalized distribution P(h) is obtained, and decision making is realized by selecting the data with the highest probability under the hypothesis with the highest probability.

Vertical schemes therefore correspond to inference under a fixed causal model and can be interpreted in Bayesian terms, consistent with the free-energy principle. On the other hand, one possible implementation of horizontal schemes is inverse Bayesian inference. Thus Bayesian inference generates cause–effect relations depending on experience in the form of Boolean algebras, while inverse Bayesian inference provides an operation that continuously adds new Bayesian inferences.

In other words, Bayesian estimation plays the role of the vertical scheme of inference, while inverse Bayesian inference plays the role of the horizontal scheme. A system that performs decision making by Bayesian inference while executing inverse Bayesian inference therefore realizes the interplay between the vertical scheme and the horizontal scheme.

The least-used hypothesis is selected because modifying a highly dominant hypothesis would strongly disrupt the current recognition structure. By contrast, modifying a weakly supported hypothesis allows the system to introduce a new causal context while preserving the overall stability of existing contexts. In this sense, inverse Bayesian inference corresponds to replacing one sub-Boolean algebra with another, thereby implementing the gluing process algebraically.

**Figure 3:**
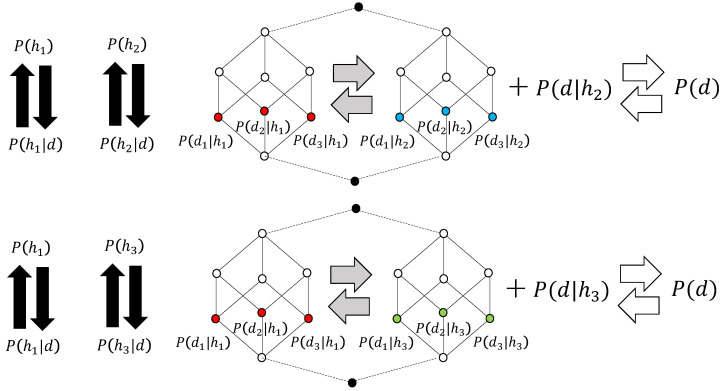
This diagram illustrates the relationship between the vertical scheme, which represents Bayesian inference, and the inverse Bayesian inference, which shows the horizontal scheme where the likelihood of a hypothesis changes depending on the distribution of the data. The Hasse diagram in the center represents a non-distributive orthomodular lattice (quantum logic) that includes the system’s hypotheses as sub-Boolean algebras, with sub-Boolean atoms represented by colored circles. Different colors (red, blue, green) represent atoms of different sub-Boolean algebras. Black circles represent the least and the greatest element, respectively. Solid lines indicate covering relation (order relation where no other elements are placed in between), while broken lines indicate that the elements are identical.

Bayesian inference generalizes conditional probabilities that hold under specific circumstances into marginal probabilities, thereby using them as units of recognition. If we consider the specific conditions as the cause of the generalization operation and the general condition as the result of generalization (the reverse operation is specialization), then P(h|d) is the cause and P(h) is the effect. Bayesian inference is an operation that repeatedly performs both operations to achieve agreement, obtaining P(h)=P(h|d). Therefore, it remains a vertical scheme. The left diagram in Figure 3 illustrates this.

The resulting hypothesis *h* is composed of the probability distribution of the likelihood, i.e., P(d|h), and the sum of these components forms the whole. Therefore, as shown in the central figure of Figure 3, a sub-Boolean algebra where P(d|h) is an atom (an element of the lattice directly above the least element) corresponds to P(h). In Figure 3, the whole is constructed from two sub-Boolean algebras, each corresponding to $P(h_{1})$ and $P(h_{2})$. In Bayesian inference, P(h) is constantly calculated within the whole, and decisions are made, thus involving movement between the two hypotheses. This appears to represent a horizontal scheme because it involves horizontal computational operations. However, this is not the case. To clarify this point, in Figure 3, movement between hypotheses within the framework of Bayesian inference is represented by gray horizontal arrows, not white horizontal arrows.

The true meaning of a horizontal scheme in the inference process is inverse Bayesian inference. This is achieved by incorporating data outside of the Bayesian inference, which is composed of multiple hypotheses, thereby changing the hypotheses. In the upper part of Figure 3, this is realized by the interaction between P(d), obtained from outside the Hasse diagram of the non-distributive orthomodular lattice (quantum logic), and its likelihood $P(d|h_{2})$, which defines the hypothesis, i.e., $P\left(d|h_{2}\right)=P\left(d\right)$. As a result, the $h_{2}$ whose likelihood has changed is renamed $h_{3}$, and the result changes as shown in the lower part of Figure 3. The change in the atom’s color from blue to green is because the likelihood of the hypothesis has changed.

### 4.2. Simulation Comparison Between Bayesian Inference and Inverse Bayesian Inference

To compare the proposed interplay between vertical and horizontal schemes with conventional adaptive Bayesian inference, we introduced a minimal prediction task in a nonstationary environment. Two inference systems were compared: (i) Bayesian inference alone, in which only the prior probability of hypotheses is updated, and (ii) Bayesian inference combined with inverse Bayesian inference, in which both the prior probability of hypotheses and the likelihood structure of hypotheses are updated.

Let $h_{k}$ (k=1,…,m) denote the hypotheses and let $d_{t}\in \left\{0,1\right\}$ denote the observed event at time *t*. Bayesian inference updates the probability of hypotheses according to

(29) $$P_{t}\left(h_{k}|d_{t}\right)=\frac{P_{t}\left(d_{t}|h_{k}\right)P_{t}\left(h_{k}\right)}{\sum_{j}P_{t}\left(d_{t}|h_{j}\right)P_{t}\left(h_{j}\right)}.$$

The updated posterior is then used as the prior probability for the next step:

(30) $$P_{t+1}\left(h_{k}\right)=P_{t}\left(h_{k}|d_{t}\right).$$

This process corresponds to the vertical scheme because the likelihood structure P(d|h) remains fixed. In contrast, inverse Bayesian inference updates the likelihood of the most relevant hypothesis according to the empirical distribution of recently observed data. If the empirical event probability within a recent window of length *W* is denoted by

(31) $${\hat{P}}_{t}\left(d=1\right)=\frac{1}{W}\sum_{i=t-W+1}^{t}d_{i},$$

then the likelihood of the selected hypothesis $h_{i}$ is rewritten as

(32) $$P_{t+1}\left(d=1|h_{i}\right)={\hat{P}}_{t}\left(d=1\right),$$

(33) $$P_{t+1}\left(d=0|h_{i}\right)=1-{\hat{P}}_{t}\left(d=1\right).$$

This corresponds to the inverse Bayesian operation proposed in Equations (25)–(28), where the empirical probability of data modifies the likelihood structure of a hypothesis itself. In contrast to Bayesian inference, where the hypothesis set remains fixed, inverse Bayesian inference modifies the likelihood structure itself and thereby introduces a horizontal transformation of the hypothesis space.

To evaluate prediction performance, we defined the predicted event probability at each time step as

(34) $${\hat{p}}_{t}=\sum_{k}P_{t}\left(h_{k}\right)P_{t}\left(d=1|h_{k}\right),$$

and measured the squared prediction error

(35) $$E_{t}={\left({\hat{p}}_{t}-d_{t}\right)}^{2}.$$

The cumulative mean squared error (cumulative MSE) was then calculated as

(36) $$\mathrm{cumMSE}\left(T\right)=\frac{1}{T}\sum_{t=1}^{T}E_{t}.$$

Two types of environmental change were examined. In the slowly varying environment, the true event probability changed gradually from 0.2 to 0.35 and then to 0.5. In the abruptly changing environment, the true event probability repeatedly switched among 0.8, 0.2, 0.65, and 0.35. The purpose of these two conditions was to compare situations in which conventional Bayesian adaptation is relatively sufficient and situations in which a fixed hypothesis structure becomes inadequate.

**Figure 4:**
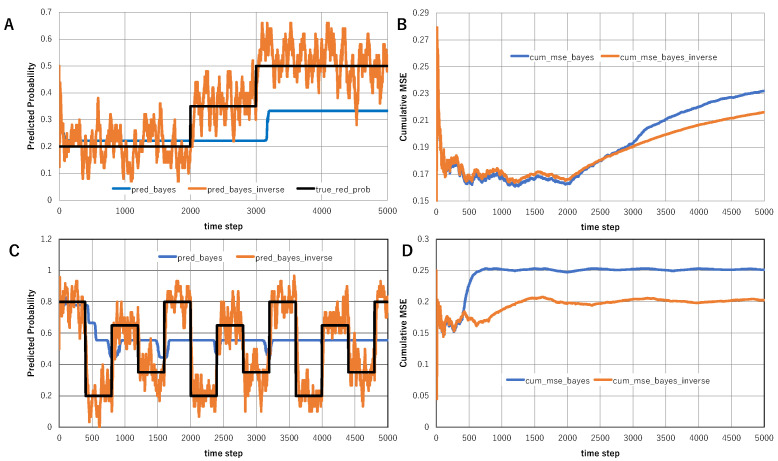
Comparison between Bayesian inference alone and Bayesian plus inverse Bayesian inference in nonstationary environments. (**A**) Slowly varying environment: comparison between the true event probability and the predictions generated by Bayesian inference alone and Bayesian plus inverse Bayesian inference. (**B**) Slowly varying environment: comparison of the cumulative mean squared error (cumulative MSE) between Bayesian inference alone and Bayesian plus inverse Bayesian inference. (**C**) Abruptly changing environment: comparison between the true event probability and the predictions generated by Bayesian inference alone and Bayesian plus inverse Bayesian inference. (**D**) Abruptly changing environment: comparison of the cumulative mean squared error (cumulative MSE) between Bayesian inference alone and Bayesian plus inverse Bayesian inference.

Figure 4A,B show the slowly varying environment. Figure 4A compares the true event probability with the predictions generated by Bayesian inference alone and Bayesian plus inverse Bayesian inference. Because the environmental change is gradual, Bayesian inference alone can adapt to some extent. However, the combination of Bayesian and inverse Bayesian inference tracks the environmental change more quickly and remains closer to the true probability. Figure 4B compares the cumulative MSE of the two methods. The Bayesian plus inverse Bayesian model maintains a consistently lower cumulative MSE than Bayesian inference alone, indicating that modifying the likelihood structure improves prediction performance even when environmental changes are slow.

Figure 4C,D show the abruptly changing environment. In this case, Bayesian inference alone adapts only slowly because the likelihood structure remains fixed. In contrast, Bayesian plus inverse Bayesian inference rapidly rewrites the likelihood of the most relevant hypothesis according to the recent empirical distribution. As a result, the predicted event probability more closely follows the true event probability after each abrupt change. The difference is particularly clear in Figure 4D, where the cumulative MSE of Bayesian plus inverse Bayesian inference remains substantially lower than that of Bayesian inference alone.

These results suggest that Bayesian inference alone is sufficient when environmental change is weak and can be approximated within a fixed hypothesis space. However, when the environment changes more strongly or when the causal structure itself becomes unstable, updating only the prior probability of hypotheses is insufficient. In such cases, inverse Bayesian inference functions as a horizontal scheme that transforms the likelihood structure itself. This provides a simple computational demonstration that the interplay between vertical and horizontal schemes improves adaptability in nonstationary environments. It also clarifies the difference between our framework and conventional adaptive Bayesian methods, which generally update probabilities within a fixed hypothesis space rather than modifying the hypothesis structure itself.

To further examine the effect of temporal smoothing in inverse Bayesian inference, we additionally introduced moving-average smoothing of the inferred event probability. In this analysis, the abrupt environmental change used in Figure 4C was retained, where the true event probability repeatedly switched among 0.8, 0.2, 0.65, and 0.35. The prediction generated by Bayesian plus inverse Bayesian inference was then smoothed by averaging over recent time steps.

More specifically, if ${\hat{p}}_{t}$ denotes the predicted event probability at time *t*, the smoothed prediction ${\bar{p}}_{t}^{\left(L\right)}$ with smoothing length *L* was defined as

(37) $${\bar{p}}_{t}^{\left(L\right)}=\frac{1}{L}\sum_{i=t-L+1}^{t}{\hat{p}}_{i}.$$

**Figure 5:**
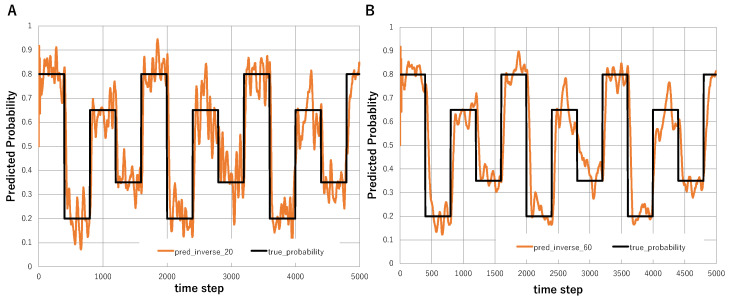
Effect of temporal smoothing on Bayesian plus inverse Bayesian inference in an abruptly changing environment. (**A**) Comparison between the true event probability and the prediction generated by Bayesian plus inverse Bayesian inference smoothed by averaging over the previous 20 time steps. (**B**) Comparison between the true event probability and the prediction generated by Bayesian plus inverse Bayesian inference smoothed by averaging over the previous 60 time steps.

Figure 5A shows the prediction smoothed over the previous 20 steps, whereas Figure 5B shows the prediction smoothed over the previous 60 steps. Shorter smoothing retains faster responsiveness to abrupt environmental changes, while longer smoothing suppresses short-term fluctuations more strongly. Thus, the 20-step smoothing preserves relatively rapid adaptation to environmental change, whereas the 60-step smoothing provides a more stable but slower response. These results illustrate the trade-off between rapid adaptation and temporal stability in inverse Bayesian inference.

## 5. Discussion

Conventional adaptive Bayesian inference, online learning, and incremental reasoning methods update parameters, prior probabilities, or hyper-parameters within a predefined hypothesis space [78,82,83,84,85,86,87]. Even when hyper-parameters are adapted, the overall structure of the model remains fixed [79,88]. In contrast, the present framework allows the likelihood structure P(d|h) itself to be rewritten through inverse Bayesian inference. Therefore, the proposed framework does not merely optimize inference within a given hypothesis space, but enables the hypothesis space itself to be transformed and expanded depending on experienced data. This property is particularly important in nonstationary environments, where the underlying causal structure may change over time [87,89]. The differences mentioned above are summarized in Table 1.

While computation is an abstract operation, computers are implemented as physical objects. Typically, computers are constructed using ultrastable materials like silicon to minimize the impact of their physical implementation. However, living organisms, while essentially biocomputers making various judgments and decisions, are composed of extremely unstable materials such as proteins, carbohydrates, and water. It is not immediately clear whether a computer composed of such unstable materials would simply be prone to errors or whether it might support qualitatively different modes of computation. Not at all. On the contrary, it is a computation open to creativity, instantly deciding to flee when danger is detected and conceiving unexpected ideas. There is no need to consider creativity or grand innovation. Biological computers, including the brain, are constantly open to creativity, suddenly sensing and judging things that are not habitual.

What exactly is judgment open to creativity? In this paper, we interpret this as a possible manifestation of the interplay between computation and matter. Unconventional computing is betting on this very point, attempting to verify it at the material level using various bio-materials, living organisms, and colloids. In contrast, this paper attempts to abstract that relationship and express the interplay between computation and matter in the form of an interplay between the computation of a vertical scheme, which is cause–effect relations, and the computation of a horizontal scheme, which is brought about by the intersection of causal relationships, and have shown that their logical structure can be understood as quantum logic generated by gluing Boolean algebras. The vertical scheme corresponds to inference under fixed cause–effect relations, and is implemented as Bayesian inference. This can be understood as a process in which the probability distribution of hypotheses is updated depending on experience and prediction error is minimized. In this sense, the vertical scheme proposed in this paper is consistent with the free-energy minimization principle proposed by Friston, which has been widely discussed as a computational principle of the brain. Free-energy minimization is a process in which internal models are updated so as to minimize the surprise of observed data, and it can be interpreted as Bayesian inference.

However, in many models based on the free-energy principle, the hypothesis space itself remains fixed, and only probability distributions within that space are updated. The horizontal scheme introduced in this paper extends this framework. The horizontal scheme is defined as a process that transforms the hypothesis space itself, and in this paper it is formulated as inverse Bayesian inference. In inverse Bayesian inference, the likelihood of hypotheses themselves is rewritten based on experienced data, thereby generating new hypothesis structures. As a result, the system does not merely optimize within the existing set of hypotheses, but continues inference while transforming the hypothesis set itself.

This structure is related to one conceptual approach to implementing the interplay between the vertical scheme and the horizontal scheme: Natural Born Intelligence (NBI) [25]. In this framework, intelligence is discussed not simply in terms of fixed computational rules, but in terms of an ongoing interaction between material processes and informational processes, through which computational structures may also be transformed.

In other words, in the closed computational systems assumed by conventional artificial intelligence, computational rules and hypothesis spaces are given in advance. In contrast, in NBI these structures themselves are updated through interactions with the environment and material processes. The horizontal scheme introduced in this paper can be understood as the computational process responsible for such updates, and inverse Bayesian inference can be regarded as its concrete implementation.

Furthermore, quantum logic generated by gluing Boolean algebras into a non-distributive orthomodular lattice provides an algebraic framework for representing such transformations of computational structures. Each Boolean algebra represents a causal structure in a particular context, and the gluing of these algebras enables transitions between contexts. In this sense, quantum logic should not be interpreted as a model that performs inference within a fixed logical system, but rather as a structure of intelligence in which the logical system itself continues to expand.

Therefore, the interplay between vertical schemes and horizontal schemes proposed in this study can be understood as a framework that integrates prediction-error minimization through Bayesian inference and the generation of hypothesis spaces through inverse Bayesian inference. This framework provides one possible theoretical basis for understanding intelligence not as a fixed computational system but as an open computational process whose structure changes through interaction with the material world, and it suggests a possible connection between computational models based on quantum logic and the concept of Natural Born Intelligence.

The framework in this paper is consistent with the theory of open quantum systems [90] when it is used for quantum-like modeling of decision making. In this interpretation, the material world corresponds to the environment. In models of decision making based on decoherence, the decision state can be described as a steady state of a quantum master equation, for example of the Gorini–Kossakowski–Sudarshan–Lindblad type. The generator of this dynamics, L, consists of two components: a Hamiltonian part *H*, corresponding to structured evolution within a given context, and a dissipative part *D*, representing the influence of the environment.

From the viewpoint of the present framework, these two components can be suggestively related to the vertical and horizontal schemes, respectively: the Hamiltonian part reflects transformations within a fixed causal structure, while the dissipative part introduces context-dependent modifications through interaction with the environment. However, it should be noted that the horizontal scheme, as introduced in this paper, refers more generally to transformations between different causal structures themselves, and is therefore not fully reduced to decoherence dynamics alone.

In this sense, decoherence can be interpreted as one mechanism that partially realizes horizontal transformations, although the latter is not limited to such processes.

## 6. Conclusions

In this paper, we formulated computation as the interplay between vertical schemes, which represent fixed cause–effect relations, and horizontal schemes, which transform those relations.

We showed that the vertical scheme corresponds to Bayesian inference, while the horizontal scheme can be implemented as inverse Bayesian inference that modifies the hypothesis space itself. This combination yields a computational process that does not remain within a fixed set of hypotheses but continuously reorganizes its own inferential structure. Furthermore, we demonstrated that this interplay can be expressed algebraically as a process of gluing Boolean algebras, resulting in a non-distributive orthomodular lattice. In this sense, quantum logic emerges not as a static logical system but as a structural consequence of the continual reconfiguration of causal contexts.

These results provide a formal framework in which inference is understood not only as optimization under given models but also as a process that generates and transforms the models themselves.

It offers a formal basis for describing open-ended computation and suggests possible applications to adaptive cognition, unconventional computing, and nonstationary environments in which the underlying causal structure itself changes over time. 

## Figures and Tables

**Table 1:** Comparison between conventional adaptive inference methods and the proposed framework.

Method	What Is Updated	Hypothesis Space	Main Limitation
Bayesian inference	Prior/posterior probabilities	Fixed	Cannot modify the likelihood structure itself
Adaptive Bayesian inference	Prior/posterior probabilities and hyperparameters	Usually fixed	Adapts weights within a predefined model class
Online learning/incremental learning	Sequential parameter updates	Fixed model or feature space	Only modifies parameters of existing models
Proposed Bayesian + inverse Bayesian inference	Prior probabilities and likelihood structure	Expandable/ transformable	Can rewrite hypothesis structures depending on experienced data

## Data Availability

The original contributions presented in this study are included in the article. Further inquiries can be directed to the corresponding author.
